# Editorial: AI-empowered services for interconnected smart plant protection systems

**DOI:** 10.3389/fpls.2024.1359627

**Published:** 2024-03-22

**Authors:** Xu Zheng

**Affiliations:** Department of Computer Science and Engineering, University of Electronic Science and Technology of China, Chengdu, China

**Keywords:** artificial intelligence, plant protection, interconnected smart system, internet of things (IoT), computer vision

The goal of our Research Topic on ‘*AI-Empowered Services for Interconnected Smart Plant Protection Systems*’ is to inspire and collect innovative and high-quality studies considering fundamental issues towards empowering interconnected smart plant protection with cutting-edge artificial intelligent techniques. This Research Topic opened on October 21, 2021, eith all final papers due by April 01, 2022. During this process, our Research Topic received a total of 14 submissions, among which six outstanding papers were accepted. These papers contribute to the development of different aspects of smart plant protection systems by considering the design of artificial intelligent models and techniques, and have shown great potential in benefiting various applications, such as the detection of fruits, disease detection for leaves, etc. Some highlights of our Research Topic are summarized below.

The Research Topic includes studies with new results on cutting-edge computer vision (CV) models for plant protection. Internet-of-Things devices like drones equipped with cameras have extended the boundary of multimedia data collection of photos and videos. Accompanying solutions that provide efficient and effective multimedia data are urgently needed. The Research Topic presents three outstanding papers on the design of deep convolutional networks for different tasks, including fruit detection and counting, disease detection for leaves, and light-weighted models designed for real-time image detection on smart devices. These studies consider the most advanced models like YOLOv5 (You Only Look Once) and Alexnet and provide refined designs to adopt the models for upstream plant protection tasks. Specifically, all authors have conducted extensive experiments to validate improved performance. For example, the model EADD-YOLO proposed in one of the papers “*EADD-YOLO: An Efficient and Accurate Disease Detector for Apple Leaf Using Improved Lightweight YOLOv5*” achieves a detection performance of 95.5% in mAP (mean Average Precision) and a speed of 625 FPS (Frames per second) on a dataset composed of images of five common apple leaf disease, which have significantly outperformed previous solutions (Zhu et al.). Meanwhile, the light-weighted model for apple leaf disease detection outperforms Alexnet by 9.31% on the accuracy of detection, while the model size is only 70% of Mobilenet, a famous light-weighted CV model. Generally, these studies extend the design of CV models for more robust and efficient solutions for smart plant protection.

The Research Topic also includes several impressive studies on AI-empowered methods for non-computer-vision tasks, including insect pest density estimation, protein ubiquitination site prediction, and secure data-sharing strategies considering the distributed data process of smart plant protection. The first study adopts readings of sensors in smart plant systems for population density estimation of brown planthoppers, showing the possibility of applying chlorophyll readings, water, silicon, and sugar for estimation. The second work provides *a Caps-Ubi model for protein ubiquitination site prediction*, with insights into cellular responses in plants and contributions on plant protection tasks (Luo et al.). The third work considers the fundamental challenge of secure data sharing in interconnected smart plant protection systems. The authors adopt the features of data distribution over smart devices to derive an efficient and secure data-sharing plan for the training of AI-empowered models. According to their results, all solutions have achieved advanced performance compared with previous state-of-the-art methods. These studies have set fundamental supports for data processing tasks in plant protection, ranging from the very basic data communication among smart devices to the sophisticated analysis of the biological characteristics used for plant protection.

Generally, our Research Topic has provided a forum sharing pioneering ideas and investigations on different aspects of smart plant protection and collected a batch of AI models specifically tailored for plant protection. To date, the Research Topic has received 1,772 downloads and 9,671 views. We believe that the Research Topic and these accepted papers provide insights for subsequent studies on cutting-edge techniques for smart plant protection.

## Author contributions

XZ: Writing – original draft, Writing – review & editing.

